# Equivalent Refractive Index Modeling and Multidomain Characterization of the Temperature Response of Loss in Fiber-Optic Macro-Bends

**DOI:** 10.3390/s26092688

**Published:** 2026-04-26

**Authors:** Haihui Shen, Dong Yang, Hu Han, Jianli Liu

**Affiliations:** 1 School of Petroleum Engineering, Yangtze University, Wuhan 430100, China; 2024730028@yangtzeu.edu.cn; 2 School of Mechanical Engineering, Yangtze University, Jingzhou 434023, China; 201972315@yangtzeu.edu.cn

**Keywords:** high temperature and high pressure (HTHP), thermo-optic effect, bending loss, numerical simulation, spectrum analysis

## Abstract

In the oil and gas industry, fiber-optic telemetry is hindered by transmission degradation from high-temperature macro-bend loss. In this study, to address the lack of a unified model, we develop a numerical framework incorporating both bending-dominated effects and thermo-optic modulation. We systematically analyze the coupled responses of multimode (MMF) and single-mode (SMF) fibers at 1.55 μm across varying temperatures (303.15~483.15 K) and bending radii (1~12 mm). Power spectral density (PSD) and phase spectra are utilized to characterize the loss response and explore its modulation mechanisms. Our results indicate that the MMF temperature response is relatively smooth, with a peak magnitude of 10^3^. In the frequency domain, increased bending raises the MMF PSD main peak by over an order of magnitude, enhancing structural features. While the MMF phase response exhibits a wide dynamic range under tight bending, it becomes unstable in weak modulation regions. Conversely, SMF exhibits more pronounced structural fluctuations (order of 10^4^) but maintains a continuous, smooth phase gradient, demonstrating superior stability. Furthermore, MMF frequency-domain characteristics are highly wavelength-dependent (1.2~2.0 μm), whereas SMF fluctuations remain below 10%, indicating a higher parameter robustness. These findings provide a theoretical foundation for optimizing downhole fiber-optic telemetry selection and signal processing strategies.

## 1. Introduction

The expansion of hydrocarbon exploration into deep and ultra-deep regimes imposes severe demands on downhole monitoring systems. Unlike legacy electronic sensors, which degrade rapidly under thermal stress, or conventional logging, which incurs high operational costs, fiber-optic sensing offers a robust solution for real-time, lifecycle data acquisition in smart well applications. Despite this potential, the practical implementation of fiber-based telemetry remains constrained by the need for long-term stability in extreme HTHP environments.

Empirical studies indicate that, in wells exceeding 4500 m, subsurface temperatures typically surpass 423.15 K, with pressures exceeding 100 MPa. In ultra-deep wells (>6000 m), these conditions escalate to over 473.15 K and 150 MPa, creating an extremely hostile environment [1]. Under such high-temperature and high-pressure (HTHP) regimes, optical fibers are prone to severe degradation, characterized by accelerated signal attenuation, compromised mechanical integrity, and reduced sensing accuracy [2]. Crucially, a synergistic effect exists between thermal exposure and pressure-induced bending stress that significantly accelerates fiber failure and represents a primary bottleneck limiting the large-scale deployment of downhole fiber-optic telemetry [3]. Consequently, elucidating the mechanisms of these environmental impacts and developing resilient material systems is of paramount theoretical and engineering importance for advancing oilfield monitoring applications.

Considering the complexities of downhole environments, investigating the thermal response of fiber-optic bending loss is essential. This research occupies a critical interdisciplinary intersection of fiber optics and materials science, examining the temperature-dependent variations in bending-induced signal attenuation. The current advancements in this field follow three primary technical trajectories: functional material integration, structural optimization of novel fibers, and the refinement of traditional sensing methodologies.

Innovation in fiber-optic sensing is currently bifurcated into functional material integration and waveguide structural engineering. In the realm of materials, researchers at East China University of Science and Technology and Southeast University exploited the elastic constant (K33) of cholesteric liquid crystals for high-sensitivity electro-thermal sensing [4]. Addressing thermal stability, a team from Jilin University developed high-entropy rare-earth niobate ceramics that overcome aging drift from 223.15 K to 1423 K [5], while scholars at the University of Illinois applied computational inverse design to program polymer thermos-mechanical properties [6]. In parallel, advancements in waveguide geometry focus on environmental robustness. Technologies such as Corning’s ClearCurve^®^ utilize trench-assisted or photonic crystal structures [7] to mitigate bending loss, ensuring signal integrity and long-term reliability in complex optical networks [8].

Conventional fiber sensing exploits the interplay between thermo-optic and bending-induced photoelastic effects. Macro-bending structures serve as a key mechanism to modulate fiber transmission, where curvature perturbations excite mode leakage and radiation. This macro-bending loss is inversely related to the bending radius [9]. Concurrently, temperature variations modify the refractive index profile, altering optical confinement and creating a measurable power–temperature response. Although reducing the bending radius enhances sensitivity—as shown by studies from Northeastern University [10]—this approach is limited by excessive insertion loss and compromised mechanical integrity. Mahabubur et al. reported that, while geometric optimization can yield sensitivities of ~0.05 dB/K, the linear dynamic range remains confined to 293.15~350.15 K [11]. Therefore, achieving an optimal trade-off between sensitivity, loss, and mechanical stability is central to advancing sensor design.

Parallel to SMF developments, MMF utilizes inter-mode coupling and radiative leakage for high-sensitivity thermal monitoring. Although constrained by modal instability, MMFs offer distinct advantages in EMI immunity and multiplexing capabilities. Notably, Wang et al. demonstrated edge filters based on cascaded heterogeneous macro-bend units for wide-range temperature sensing [12]. In structural health monitoring (SHM), MMFs provide a durable solution for thermal stress analysis and early fire detection [13]. Moreover, advances in structural design and packaging are transforming this technology into a universal platform for decoupled multi-parameter sensing, such as simultaneous strain and refractive index detection [14].

Current studies investigating the temperature response of macro-bending loss have largely centered on structural design and experimental validation, and are generally restricted to narrow temperature regimes around room temperature. As a result, the established methodologies remain optimized for benign applications with moderate performance requirements, such as environmental sensing and SHM, rather than the extreme conditions encountered in deep-earth exploration.

Despite the complexities of modeling thermal responses in extreme deep-well conditions, numerical simulation techniques for optical fibers have matured significantly. In terms of thermo-optic interactions, strategies such as the decoupled eigenmode analysis by Wisal et al. [15] have successfully clarified the mechanism of mode coupling and power oscillation driven by radial temperature gradients. Concurrently, bending loss modeling has progressed from classical Marcuse equations [16] to sophisticated full-vectorial finite element method (FEM) analysis [17]. This shift has enabled a precise correlation between macro-bending loss and key parameters like the normalized frequency (*V*). Extending beyond conventional fibers, FEM also serves as a critical tool in the development of microstructured optical fibers (MOFs), enabling the optimization of photonic crystal fibers (PCFs) [18] and hollow-core anti-resonant fibers (HC-ARFs) [19] for ultra-low loss operation in the 1.55 μm band.

While the aforementioned research demonstrates that fiber mode analysis has reached a high level of maturity under standard conditions, existing single-physics models remain inadequate for the unique challenges of deep-well HPHT environments. Specifically, these models fail to accurately capture the evolution of bending loss driven by synergistic thermo-mechanical effects. Consequently, establishing a systematic theoretical framework based on thermo-mechanical–optical multiphysics coupling is critical for addressing the current gap in mechanistic modeling. Future research necessitates the integration of multiphysics numerical simulations with in situ experimental data to derive dynamic response models tailored for extreme deep-earth conditions. Such advancements will provide a robust theoretical foundation for the deployment of fiber-optic telemetry in deep oil, gas, and geothermal exploration.

As a foundational step, we primarily focus on steady-state analysis in this study. Integrating wave optics and elastic scattering theories, we employ FEA to develop a numerical model for the thermal response of macro-bending loss in downhole environments. By incorporating macro-bending and temperature variables into the refractive index profile, we investigate macro-bending loss responses to temperature fluctuations under extreme conditions across parameter, frequency, and phase domains. We elucidate the modulation mechanism of the refractive index distribution under coupled thermal stress and geometric deformation, and furthermore identify the nonlinear evolution of bending loss relative to the downhole temperature gradient. Our findings provide a theoretical foundation for optimizing signal compensation algorithms in distributed fiber-optic sensing (DFOS) systems and facilitate the development of robust optical fiber telemetry systems.

## 2. Principles of Temperature-Dependent Bending Loss in Optical Fibers

The impact of thermal exposure on optical fibers is multifaceted, affecting transmission, mechanics, and material structure. Transmission loss is primarily governed by the Thermo-Optic Effect (TOE), and stress-induced scattering arises from the coefficient of thermal expansion (CTE) mismatch between fiber layers [20]. Empirical data suggest a critical threshold at 423.15 K, beyond which attenuation increases exponentially. Mechanically, thermal loading accelerates stress corrosion and fatigue, diminishing tensile strength while amplifying macro-bend sensitivity [21]. At the structural level, high temperatures induce the outgassing of coating materials, forming microporous defects. Temperatures exceeding 623 K ultimately trigger structural relaxation within the silica matrix, causing permanent waveguide failure [22].

**Figure 1:**
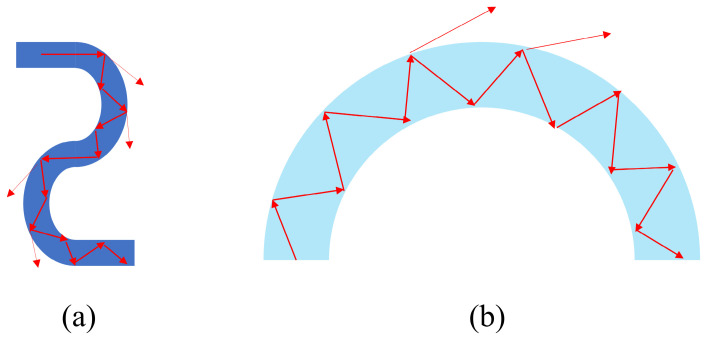
Schematic diagram of fiber bending: (**a**) micro-bend; (**b**) macro-bend.

In high-pressure regimes, fiber performance is governed by external mechanical stresses. Hydrostatic pressure acts upon the fiber to induce geometric micro-deformations, resulting in macro- or micro-bending. This deformation modulates the refractive index profile and induces significant birefringence [23], as shown in Figure 1. The study of these mechanisms is pivotal for distributed sensing in the petroleum and geological sectors, where bending is a key driver of signal failure [24]. Mechanistically, bending causes the leakage of optical modes, resulting in attenuation and a sharp decline in the signal-to-noise ratio (SNR). In engineering contexts, a low SNR risks the missed detection of critical events [25]. Thus, characterizing fiber bending behavior is indispensable for improving sensor longevity, refining packaging techniques, and achieving precise signal interpretation [26].

### 2.1. Principles of Fiber-Optic Bending

**Figure 2:**
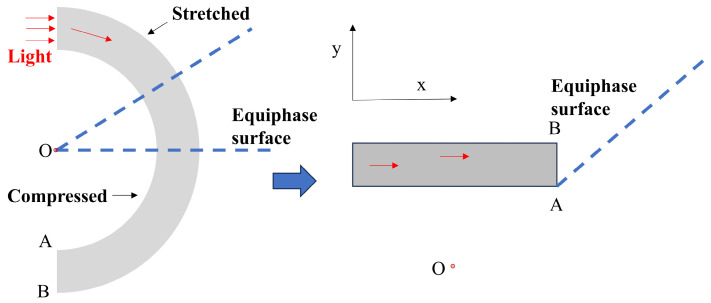
The conformal mapping of a bent fiber.

In high-pressure subsurface environments, the micro-bending disturbance mechanism in fiber-optic sensing systems involves complex multiphysics coupling. At its core, external hydrostatic [27] and non-uniform confining pressures are transmitted to the quartz core through structural mechanical pathways. These pressures form a non-uniform radial stress field [28] modulated by material property mismatches, which ultimately induces micro-bending deformation with distinct spatial spectral characteristics. For a fiber undergoing in-plane bending, propagation characteristics are described using the equivalent refractive index method. As illustrated in Figure 2, the fiber axis is assumed to lie within the bending plane with a radius *R* and a center of curvature outside the cross-section. A local Cartesian coordinate system is established, with the *x*-axis aligned with the axial direction of the straight fiber. The *y*-axis points radially away from the center of curvature, where *y* = 0 corresponds to the fiber’s geometric centerline. After bending, the geometric path length increases in the outer region (*y* < 0), whereas the path in the inner region (*y* > 0) shortens, leading to differential phase accumulation at various radial positions.

At low curvature, where the bending radius *R* significantly exceeds the fiber’s lateral dimensions, conformal mapping can transform a curved waveguide into an equivalent straight waveguide. However, the refractive index distribution requires a geometric correction factor [29]. This equivalence transformation is governed by the principle of phase conservation. Specifically, the propagation constant remains invariant between the curved and equivalent straight coordinate systems. Consequently, the equivalent refractive index at any radial position within the bent fiber satisfies the following first-order approximation:

(1) $$n(x,y)=n_{0}\exp(\frac{y}{R})\approx n_{0}(1+\frac{y}{R})$$

Here, *n*_0_ denotes the radial refractive index distribution of the straight optical fiber. This expression reflects the impact of geometric curvature on the local propagation constant. Specifically, the equivalent refractive index decreases in the outer region (*y* < 0) and increases in the inner region (*y* > 0), creating a supplemental refractive index gradient in the transverse direction. This gradient modifies the mode field distribution and intermodal coupling conditions, establishing the geometric foundation for bending loss. Notably, the validity of this equivalence relationship relies on several key assumptions: single-plane bending, adiabatic curvature variations, and the neglect of higher-order curvature terms and stress-induced refractive index perturbations.

The second mechanism influencing bent fibers is the stress-induced modulation of the refractive index, governed by the elasto-optic effect. Upon bending, the fiber experiences differential mechanical loading: the outer region is subjected to tension, while the inner region undergoes compression, altering the refractive index distribution [25]. Specifically, the outer curvature (Side B) experiences tensile strain (*ξ* > 0). According to elasto-optic theory, this tension typically induces a reduction in the refractive index. Conversely, the inner curvature (Side A) is subjected to compressive strain (*ξ* < 0), which results in an increase in the refractive index. Consequently, the perturbation in the fiber’s refractive index is expressed as follows:

(2) $$\Delta n=n_{i}-n_{0}=-C_{2}\sigma_{i}-C_{1}(\sigma_{j}+\sigma_{k})$$

Here, *σ*_i,j,k_ represent the components of the stress tensor, while *C*_1_ and *C*_2_ denote the first and second stress-optic coefficients, respectively. For silica optical fibers, these coefficients are approximately *C*_1_ ≈ 4.22 × 10^−12^ Pa^−1^ and *C*_2_ ≈ 0.65 × 10^−12^ Pa^−1^. Under bending conditions, the primary strain occurs along the axial (*z*) direction and is defined as *ξ*_z_ = *y*/*R*, where *y* is the radial distance from the neutral axis. Applying the isotropic Hooke’s law, the stress components are expressed as *σ_z_* = *C*_11_*ξ_z_* and *σ_x_* = *σ_y_* = *C*_12_*ξ_z_*. For silica fiber, the elastic stiffness constants are *C*_11_ ≈ 86.3 GPa and *C*_12_ ≈ 10.3 GPa [17]. Consequently, the stress-induced refractive index perturbation, *n*_s_(*x*,*y*), is given by the following:

(3) $$n_{s}(x,y)=n_{0}+△n(x,y)=n_{0}-\frac{y}{R}[C_{2}C_{12}+C_{1}(C_{12}+C_{11})]$$

The stress-induced anisotropic refractive index, after neglecting higher-order terms, is thus defined as follows:

(4) $$n(x,y)=n_{s}(x,y)\times (1+\frac{y}{R})\approx n_{0}(1+\frac{y}{R}[1-\frac{1}{n_{0}}(C_{2}C_{12}+C_{1}(C_{12}+C_{11}))])$$

We rewrite the above expression to align with Equation (1), and let

(5) $$\frac{1}{R_{\mathrm{eff}}}=\frac{1}{R}[1-\frac{1}{n_{0}}(C_{2}C_{12}+C_{1}(C_{12}+C_{11}))])$$

After simplification, the above expression yields

(6) $$R_{\mathrm{eff}}\approx \frac{n_{0}R}{n_{0}-0.414}$$

The correction for the elasto-optic effect is predicated on the assumption of material homogeneity (fused silica), while the influence of the coating is neglected due to the lack of specific material parameters. Consequently, the effective bending radius, *R*_eff_, is applicable across the entire fiber cross-section. Furthermore, as this correction is derived solely from the intrinsic physical properties of the silica matrix, it is independent of modal distribution. Thus, the theoretical value of *R*_eff_ is identical for both SMF and MMF. By approximating the refractive index of silica as *n*_0_ ≈ 1.45 [30] and applying Equation (6), we adopt a value of *R*_eff_ ≈ 1.40 *R*. This result indicates that the elasto-optic effect effectively increases the bending radius (or reduces the effective curvature), thereby mitigating bending loss.

Considering both the geometric and stress-optic effects induced by fiber bending, the comprehensive expression for the resultant refractive index change is derived as follows:

(7) $$n(x,y)=n_{0}(1+\frac{y}{1.4R})$$

In this formulation, *n*_0_ denotes the intrinsic refractive index, and *y*/(1.4*R*) serves as the bending-induced perturbation term. This transformation maps the complex three-dimensional bending problem onto an equivalent straight fiber model with a tilted index profile. Consequently, computational complexity is drastically reduced, facilitating efficient numerical modeling.

Although we employ a 2D cross-sectional approximation in this study, this approach relies on the adiabatic assumption that longitudinal curvature variations remain negligible, facilitating a focused analysis of the multidomain coupling between thermal perturbations and modal leakage, which remains the primary cause of total loss under stable macro-bending conditions.

### 2.2. Bending Principles of Optical Fibers Under High-Temperature Conditions

For high-temperature well applications, fiber loss stems from the superposition of thermally induced attenuation and mechanical macro-bending effects. Crucially, the synergistic interaction between these two mechanisms drives the total signal degradation and forms the core of this investigation.

#### 2.2.1. Principles of High-Temperature Attenuation in Optical Fibers

Downhole temperatures increase linearly with depth, following a standard geothermal gradient of ~3 K/100 m barring local thermal anomalies. This results in extreme conditions for deep wells, where temperatures often surpass 423.15 K or even 473.15 K [31]. In this regime, the fiber’s refractive index is governed by thermo-optic mechanisms—specifically, the combined contribution of thermal expansion and electronic polarizability—as described by the following:

(8) $$\frac{dn}{dT}=-{\left(\frac{\rho \partial n}{\partial \rho }\right)}_{T}\gamma +{\left(\frac{\partial n}{\partial T}\right)}_{\rho },$$

In this context, *ρ* is the density and *γ* is the coefficient of thermal expansion. The right-hand side separates the index modulation into density-driven (first term) and polarizability-driven (second term) components [21]. Since the contribution from thermal expansion (10^−7^) is two orders of magnitude smaller than that of polarizability (10^−5^) in silica fibers, the former can be disregarded. Thus, the thermo-optic response is dominated by polarizability, leading to the simplified thermo-optic coefficient (TOC) d*n*/d*T* in Equation (9). Here, *δ* = 2.0 × 10^−5^ K^−1^ is the thermal coefficient of polarizability, and *n* denotes the local refractive index. The resulting temperature-dependent index profile is described by Equation (10), with a reference temperature *T*_0_ = 293.15 K.

(9) $$\frac{dn}{dT}=\frac{\left(n^{2}-1)(n^{2}+2\right)}{6n}\cdot \delta$$

(10) $$n(T)=n_{0}+\frac{dn}{dT}(T-T_{0})$$

#### 2.2.2. The Combined Effects of Bending and Elevated Temperature on the Refractive Index of Optical Fibers

Mechanical bending perturbs the cylindrical symmetry of the waveguide, facilitating the coupling of guided core modes to radiation modes in the cladding, which results in energy leakage. Crucially, the magnitude of this bending loss exhibits a strong, nonlinear dependence on the bend radius, *R* [32], while temperature-induced attenuation is governed primarily by the TOE. Thermal fluctuations modulate the refractive indices of both the core and cladding materials, thereby directly altering fundamental waveguide parameters such as the normalized frequency $V=k_{0}a\sqrt{{n_{\mathrm{core}}}^{2}-{n_{\mathrm{clad}}}^{2}}$ [33].

According to Marcuse’s theory, the bending attenuation coefficient for an optical fiber with a bend radius *R* can be expressed as follows:

(11) $$2\alpha_{C}=\frac{\sqrt{\pi }U^{2}}{e_{i}W^{3/2}\sqrt{aR}V^{2}K_{i-1}(W)K_{i+1}(W)}\exp(-\frac{2W^{3}}{3a^{2}\beta^{2}}\frac{R}{a})$$

Here, $U=a\sqrt{k^{2}n_{\mathrm{core}}^{2}-\beta }$ is the normalized phase parameter, $W=a\sqrt{\beta^{2}-k^{2}n_{\mathrm{clad}}^{2}}$ is the radial normalization parameter, k=2π/λ is the vacuum wavenumber, *a* is the core radius, and *β* is the modal propagation constant. The term *K*_i_ denotes the i-th-order modified Bessel function, associated with a coefficient *e*_i_ (where *e*_i_ = 2 for i = 0, and *e*_i_ = 1 for i ≠ 0). Therefore, any temperature fluctuation that modifies the refractive index will inherently shift the operating point defined by *U* and *W*, and the normalized frequency *V*.

Additive noise is defined as the linear summation of independent noise sources, primarily thermal and shot noise, with the optical signal. However, attenuation arising from thermo-mechanical coupling in bent fibers represents a multiplicative or parametric effect rather than additive noise. Here, temperature and bending synergistically alter the refractive index distribution, creating anisotropic perturbations. The thermal effect is intrinsic to the bending loss mechanism [34], where temperature acts as a governing parameter that shifts the fundamental exponential bending loss function. By altering the refractive index, temperature modulates the loss magnitude, and thus the unified material refractive index model under these coupled effects is expressed as follows:

(12) $$n_{{\mathrm{sio}}_{2}}(x,y,T)=n_{0}(1+\frac{dn}{dT}(T-T_{0})+\frac{y}{R_{\mathrm{eff}}})$$

Though Equation (11) presumes weak guidance, it still holds for the MMF analyzed here, since the relative index difference Δ(~1.5%) falls within the standard bounds of the approximation. Beyond that, this analytical form faithfully captures the regular modulation of the loss function by temperature-induced parameter shifts—a behavior corroborated by our full-wave simulations.

Our approach in this study does not account for the mechanical stress or thermal expansion effects of the fiber coating. In HTHP downhole environments, the CTE mismatch between the coating and the silica substrate often drives long-term sensor drift and mechanical failure. However, this model focuses on fundamental physics to clarify the dominant role of the TOE in the topological evolution of macro-bend loss. Because the TOC of silica (~10^−5^ K^−1^) dominates the magnitude of refractive index variations, a bare-fiber model provides a clear physical benchmark for understanding signal attenuation under extreme conditions. Future research should incorporate a multiphysics framework—integrating mechanical, thermal, and optical fields—to quantitatively assess the impact of coating stress-induced birefringence on sensing reliability.

## 3. Model Development

**Figure 3:**
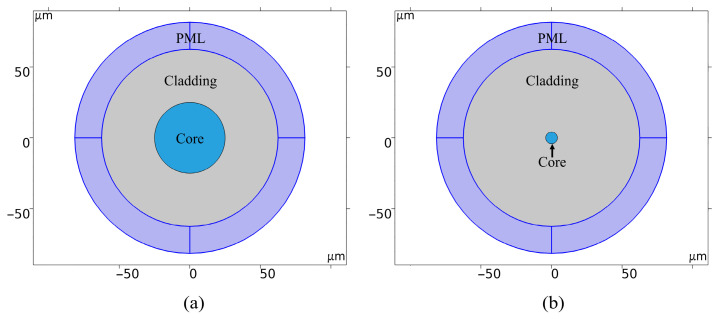
The 2D geometric models of optical fibers: (**a**) MMF; (**b**) SMF.

In this study, we employ COMSOL6.3 (Wave Optics Module) to develop a frequency-domain electromagnetic model of an optical fiber cross-section, facilitating the quantitative analysis of thermal response characteristics under micro-bending conditions. Geometric parameters are derived from Table 1, with the cross-sectional structure illustrated in Figure 3. The model utilizes eigenmode analysis as the solution type. By calculating the complex effective refractive index, *n*_eff_ = *n*′ + i*n*″, the radiation loss coefficient, *α* = 2*k*_0_*n*″ (where *k*_0_ is the vacuum wavenumber), is derived. Thermal and equivalent curvature perturbations are introduced via a parameter sweep to determine the loss–temperature relationship, with the equivalent refractive index defined according to Equation (12).

Previous research [35] has indicated that, during the calculation of bending or thermal losses, the core-guided mode and the cladding whispering gallery mode (WGM) undergo coupling interference. This interference—either constructive or destructive—depends on the wavelength, bending radius, and cladding refractive index. Thermal variations trigger thermo-optic and thermal expansion effects that alter these parameters, significantly shifting the coupling state between the core mode and the WGM. Such oscillatory, nonlinear responses are detrimental to optical transmission and sensing systems requiring stable output, as they complicate calibration and induce sensitivity instability within narrow temperature ranges. Furthermore, as this study prioritizes intrinsic optical coupling and thermal parameter evolution over mechanical load transfer, cladding mechanical effects are characterized by equivalent stress-optic terms and equivalent curvature parameters. Consequently, the present model neglects secondary cladding influences. An open-boundary formulation is implemented, incorporating a perfectly matched layer (PML) at the periphery. Calculations are performed on a 2D cross-section, applying a uniform axial approximation with a 1 m default thickness. This approach is appropriate for micro-bending scenarios with adiabatic axial curvature variations. This framework ensures physical interpretability while enabling reproducible, convergent calculations of coupled thermal and micro-bending losses.

### 3.1. The Parameters of Models

Table 1 lists the model parameters. For SMF, the germanium-doped core has a refractive index of 1.44~1.47, whereas the cladding—comprising nearly pure silica—ranges from 1.44 to 1.46. MMF cladding is similar to that of SMF, with a core refractive index ranging from 1.47 to 1.49 [36]. This study adopts an ideal step-index refractive index profile as the reference model to obtain mode field solutions with well-defined physical boundaries. Compared to gradient-index structures, this model eliminates interference from additional mode coupling degrees of freedom. This more clearly reveals the intrinsic relationship between macro-bending radiation loss and thermo-optic modulation.

The model operates within a temperature range of 303.15~483.15 K to simulate conditions in deep to ultra-deep downhole environments. In ultra-deep wells, hydrostatic pressures exceeding 150 MPa exerted through non-uniform casing often induce micrometer- to millimeter-scale fiber buckling [37]. This study simplifies this complex high-pressure behavior into a characteristic bending radius *R* within a confined space. By scanning *R* from 1 to 12 mm, the model encompasses conditions ranging from conventional deployments to extreme pressure-induced buckling. The TOCs, determined via Equation (9), indirectly compensate for pressure loads. Both MMF and SMF cladding TOCs are 1.0 × 10^−5^ K^−1^, while the core coefficients are 1.5 × 10^−5^ K^−1^ for MMF and 1.2 × 10^−5^ K^−1^ for SMF.

Experimental characterization is central to validating the TOE. For instance, the Tsukuba Electronics and Communications Laboratory analyzed GeO_2_-doped silica glass between 613 K and 808 K, reporting a TOC of approximately 1.3 × 10^−5^ K^−1^~1.4 × 10^−5^ K^−1^ at a wavelength of 0.5894 μm [38]. Similarly, researchers from the University of Electronic Science and Technology of China investigated step-index fibers with a GeO_2_-doped core and pure silica cladding, measuring a TOC of 1.178 × 10^−5^ K^−1^ [39]. Furthermore, a study by the Beijing Institute of Technology indicated that, for silica fibers operating over a wide temperature range (0~1273 K), the TOC varies between 1.09 × 10^−5^ K^−1^~1.417 × 10^−5^ K^−1^ [40]. These empirical data align closely with the theoretical results derived in this work, providing strong validation for the thermo-optic analysis.

Although the TOC of silica glass exhibits slight temperature dependence over wide ranges, linear approximations (*d*_1_, *d*_2_, *d*_3_) are employed because variations remain within a minimal margin (under 10%) up to 483 K. This approach is consistent with established empirical data [38,39,40]. By adopting these approximations, the model ensures a balance between computational efficiency and physical accuracy in characterizing the steady-state thermal response.

### 3.2. The Model Boundary Conditions and Mesh

**Figure 4:**
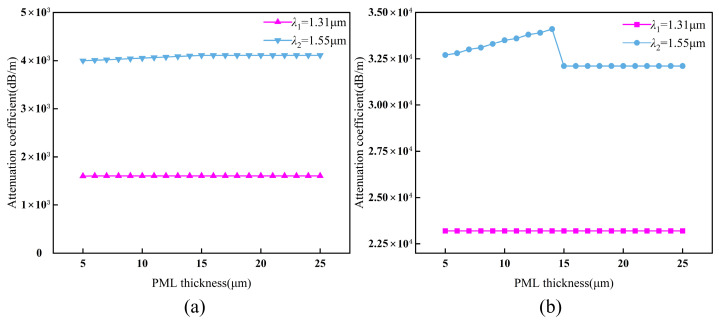
The effect of PML thickness on fiber model attenuation coefficient at two operating wavelengths: (**a**) MMF; (**b**) SMF.

In the open-end model, a PML is applied to the outer surface of the fiber cladding. A scattering boundary is further integrated at the PML’s outer limit to suppress spurious reflections in the frequency domain. Since the PML is an anisotropic region characterized by a complex electric field, its thickness directly influences calculation accuracy within the COMSOL environment. While its real refractive index matches the adjacent cladding, the imaginary component and layer thickness govern modal power loss within this region. Mode radiation reaches its maximum at a bending radius of *R* = 1 mm. Figure 4 illustrates the influence of PML thickness on mode loss across two operating wavelengths (*λ*). The relative rate of change for PML thicknesses between 15 μm and 24 μm is denoted by *ε*. For MMF, the observed values are *ε*_M,1.31_ ≈ 3% and *ε*_M,1.55_ ≈ 2%. Correspondingly, for SMF, the rates are *ε*_S,1.31_ ≈ 2% and *ε*_S,1.55_ ≈ 1%. In finite element analysis (FEA), a relative change rate below 5% in key responses—such as field strength or scattering parameters—indicates numerical convergence. Under such conditions, the PML is considered sufficiently thick, as further increases yield diminishing returns in accuracy [41]. Consequently, the imaginary part of the effective refractive index converges at a PML thickness of 15 μm, which is maintained for all subsequent simulations.

**Figure 5:**
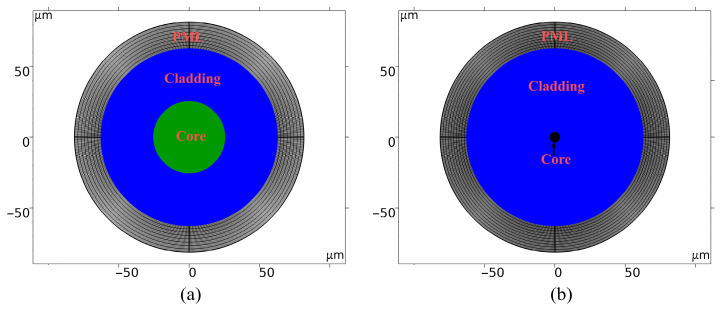
The model mesh partitioning: (**a**) MMF; (**b**) SMF (the cores are in green/black and cladding is in blue, while the PML is gray).

**Figure 6:**
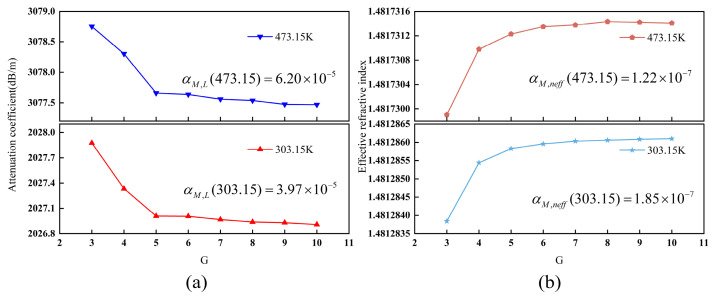
The convergence behavior of the MMF model under different *G*: (**a**) the relationship between *G* and the attenuation coefficient; (**b**) the relationship between *G* and the effective refractive index.

In FEA, mesh quality is critical to the accuracy of numerical results. Effective modeling requires a meshing strategy that balances computational efficiency with a sufficiently high mesh density to capture structural details. This study adopts the meshing strategy illustrated in Figure 5. To resolve field variations across the fiber cross-section, unstructured meshes are applied to the core and cladding, whereas a structured mesh is utilized for the PML. Furthermore, because signals propagate through the core and the fiber bends toward the positive *y*-axis, the core and upper cladding require high-density meshes to accurately resolve mode field profiles. The maximum mesh size is defined as *λ*/*G*, where *G* represents the mesh parameter. Conversely, the lower cladding is relatively sparse, with a maximum mesh size of *λ*/3. The PML incorporates a 15-layer mapped structured mesh to effectively suppress spurious reflections. Figure 6 illustrates the convergence of the MMF model as a function of *G*, with *G* = 10 serving as the reference point. As shown in Figure 6a, for *G* ≥ 5, the relative change rates of the attenuation coefficients at 303.15 K and 473.15 K converge to 3.97 × 10^−5^ and 6.20 × 10^−5^, respectively. This indicates that further mesh refinement yields negligible improvements. Similarly, Figure 6b shows that the effective refractive index stabilizes for *G* ≥ 5, with change rates at 303.15 K and 473.15 K reaching 1.85 × 10^−7^ and 1.22 × 10^−7^, respectively. These results confirm that the model achieves grid independence at *G* = 5.

**Figure 7:**
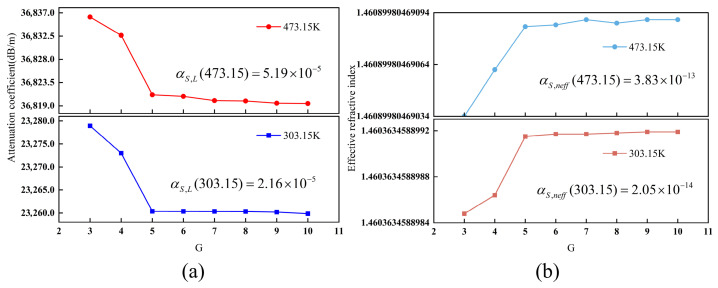
The convergence behavior of the SMF model under different *G*: (**a**) the relationship between *G* and the attenuation coefficient; (**b**) the relationship between *G* and the effective refractive index.

The convergence of the SMF model with respect to the mesh parameter *G* is analyzed in Figure 7. For G ≥ 5, the relative deviation of the attenuation coefficient stabilizes at approximately 2.16 × 10^−5^ (Figure 7a) at 303.15 K, while the effective refractive index shows negligible variations below 10^−12^ (Figure 7b). Specifically, at 473.15 K, the deviations are 5.19 × 10^−5^ and 3.83 × 10^−13^, respectively. Based on these convergence metrics, *G* = 5 was selected for all ensuing calculations.

### 3.3. Verification of the Model’s Physical Consistency

**Figure 8:**
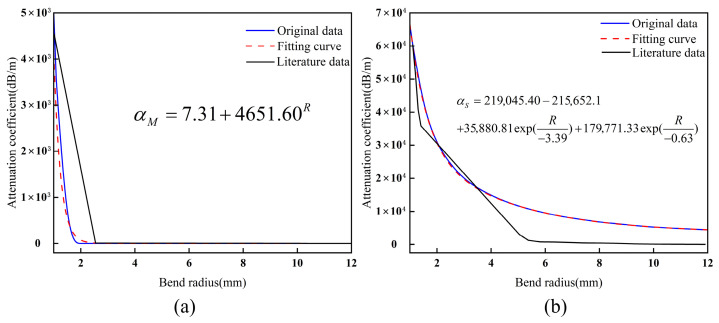
The trend of fiber attenuation coefficients with *R*: (**a**) the trend of MMF attenuation coefficient with *R*; (**b**) the trend of SMF attenuation coefficient with *R*.

Utilizing the refractive index distribution from Equation (7) and the parameters in Table 1, Figure 8 illustrates the evolution of MMF and SMF attenuation coefficients as a function of the bending radius *R* at 1.55 μm. The simulation results (blue line) indicate that decreasing *R* weakens the waveguide’s light-field confinement, resulting in an exponential increase in radiation loss. This trend aligns closely with the exponential fitting model (red dashed line, *R*^2^ > 0.99). The comparison with experimental data [25,35] shows consistent macroscopic trends, with peak errors at minimal bending radii limited to 5% for MMF and 3% for SMF. This strongly validates the model’s effectiveness in predicting limiting losses under extreme bending. However, in the descending slope and “tail” regions of the attenuation curve, the simulation results deviate slightly from the experimental data due to simplified physical assumptions.

For MMF (Figure 8a), the simulated loss cutoff characteristics are steeper than the experimental observations. This discrepancy arises from the model’s reliance on an ideal step-index refractive index profile, whereas the reference experiments primarily utilized graded-index fibers, whose continuous refractive index gradient enhances inter-mode coupling and energy redistribution. This results in the gradual attenuation of higher-order mode (HOM) radiation and a more pronounced “tail” feature at small bending radii [42]. Consequently, while the current model effectively represents limiting loss behavior under tight bending, it simplifies the gradual mode coupling processes inherent in graded-index structures. This limitation defines the applicable scope of the subsequent frequency-domain and phase analysis.

For SMF (Figure 8b), the simulated losses are slightly higher than the experimental data at larger bending radii. This discrepancy stems partly from the dynamic range and noise floor of the measurement system, which induce data compression within the low-loss regime. Additionally, the current model does not explicitly account for the fiber coating. In practice, the coating absorbs and confines radiation modes, thereby marginally reducing effective loss. Collectively, these factors explain the deviation between the simulation and experiment results in the low-loss regime.

Overall, the proposed model successfully captures the dominant influence of the bending radius on fiber loss within a unified framework. Its predicted trends and magnitudes remain consistent with experimental observations. However, this model primarily aims to elucidate the fundamental physical principles of coupled thermal and bending effects. Its predictive accuracy for graded-index multimode fibers and complex engineering structures requires further expansion and experimental validation.

**Figure 9:**
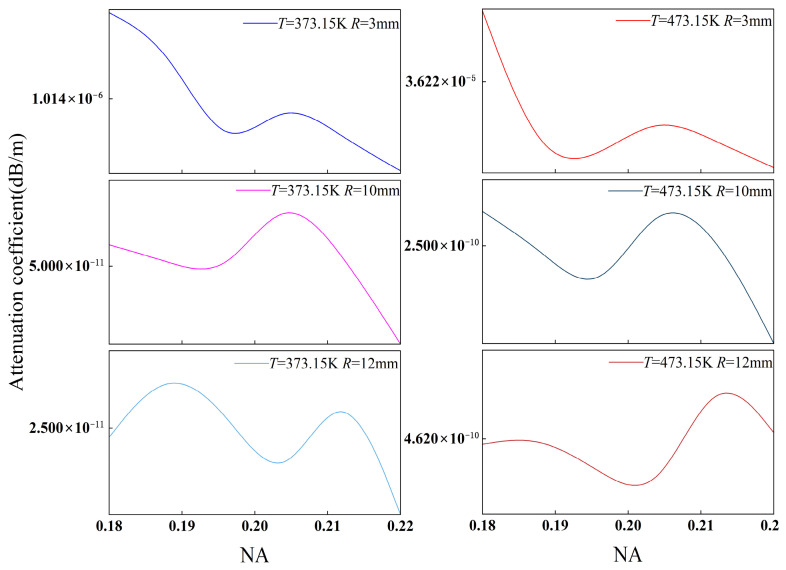
The trend in the attenuation coefficient of MMF as a function of NA at different temperatures and bending radii.

**Figure 10:**
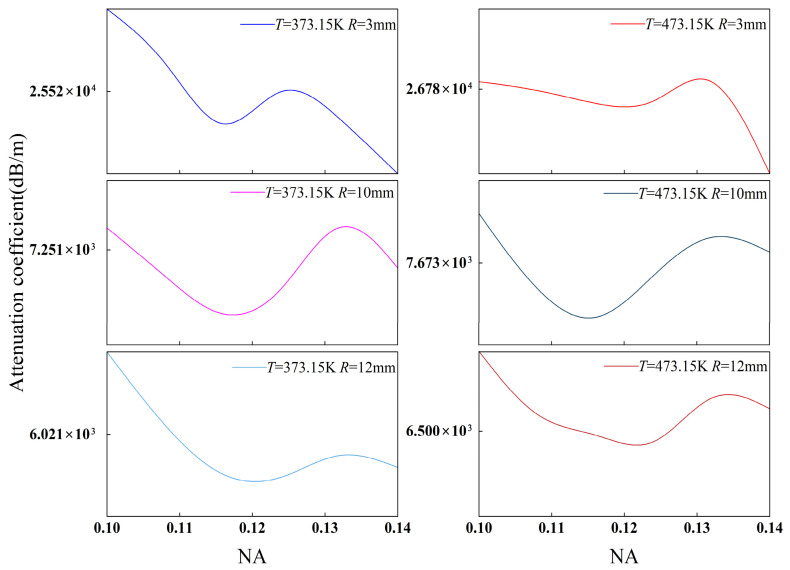
The trend in the attenuation coefficient of SMF as a function of NA at different temperatures and bending radii.

To verify the model’s physical validity, we investigated the combined effects of the bending radius and temperature. Figure 9 and Figure 10 illustrate the correlation between the attenuation coefficient and the numerical aperture (NA), with the NA ranging from 0.1 to 0.14 for SMF and 0.18 to 0.22 for MMF [43]. Our results show that the attenuation coefficients of both fibers exhibit a robust parametric dependence on the NA, regardless of temperature fluctuations. As the NA increases, the core–cladding refractive index contrast rises, strengthening modal confinement within the core. This consequently suppresses the probability of guided-to-radiation mode coupling under bending conditions, resulting in an overall decrease or minor fluctuation in bending loss. This trend aligns with classical bending loss theory, indicating that the model accurately captures the influence of waveguide structural parameters on loss.

Bending radius variations constitute the most significant source of loss modulation. Increasing the bending radius from 3 mm to 12 mm leads to a pronounced reduction in the attenuation coefficients for both MMF and SMF. The underlying mechanism involves the curvature-induced regulation of modal stability. Specifically, a smaller bending radius increases the waveguide curvature, which lowers the effective refractive index barrier of the guided mode, facilitating the leakage of modal energy into the cladding, while a larger radius conversely enhances confinement. Despite local fluctuations at varying NAs, the dominant trend dictated by the bending radius remains consistent, validating the model’s ability to characterize the decisive influence of curvature on modal stability.

Under identical bending and temperature conditions, the SMF attenuation coefficient is significantly higher than that of the MMF. This is attributed to the relatively diffuse mode field distribution in SMFs, where the fundamental mode is susceptible to evanescent wave leakage through the weakened potential barrier under tight bending. Conversely, the larger core and higher NA of MMFs ensure that low-order mode groups remain strongly confined within the core, enhancing their robustness against bending-induced perturbations. Furthermore, the MMF demonstrates more pronounced non-monotonic fluctuations with increasing NA, reflecting the complex impact of intermodal interference on loss modulation.

Temperature influence is primarily mediated by the TOE. Thermally induced changes in the refractive index of silica subsequently modulate the effective mode index (*n*_eff_) and the mode field integral. As illustrated in Figure 9 and Figure 10, rising temperature primarily manifests as a lateral shift in the absolute loss, while the evolutionary framework defined by the *R* and NA remains consistent. This confirms the model’s robustness, specifically its ability to distinguish between structure-dominated “trend terms” and temperature-induced “perturbation terms”.

As illustrated in Figure 9 and Figure 10, the observed trends in NA sensitivity, the dominance of the bending radius, and the magnitude of temperature modulation are consistent with theoretical predictions for fiber bending loss. This suggests that the model effectively replicates the primary physical mechanisms governing fiber bending loss at elevated temperatures, thereby validating its robustness and suitability for downhole sensing applications.

To provide a clear boundary for the proposed model and prevent over-extrapolation, it is necessary to explicitly outline its assumptions and simplifications. Primarily, this remains a static (steady–state) model that assesses thermal response and loss traits at discrete equilibrium states via parameter sweeps—thus ignoring transient thermo–mechanical coupling along with time–dependent dynamic behaviors. Placing intrinsic optical coupling mechanics ahead of complex physical load transfers, the model adopts equivalent stress-optic terms and equivalent curvature parameters. Hence, we omit dynamic mechanical loads and secondary cladding mechanical effects. Furthermore, simulations run on a 2D cross–section using a uniform axial approximation. Such geometric simplification presumes adiabatic axial curvature variations along with single–plane bending—a premise that may not wholly represent the three–dimensional complex spatial deformations found in real downhole environments. These simplifications are deliberately chosen to secure computational convergence and physical interpretability for static macro–bending cases.

## 4. Simulation Analysis and Discussion

### 4.1. Electromagnetic Field Distribution in Bent Optical Fibers at Elevated Temperatures

**Figure 11:**
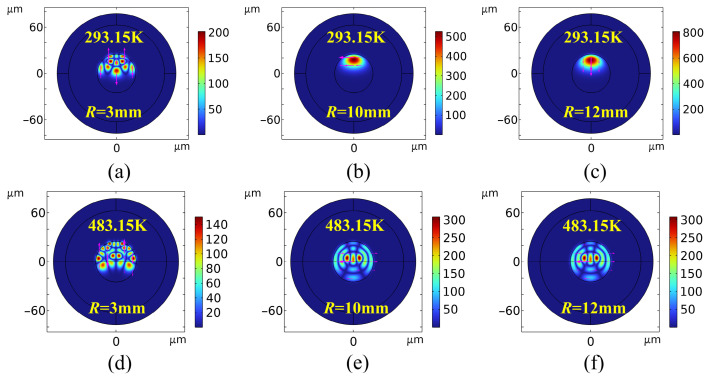
A comparison of electromagnetic field maps for bending losses in MMF at room temperature and at 483.15 K (the color intensity represents the electric field magnitude, and magenta arrows indicate magnetic field vectors).

**Figure 12:**
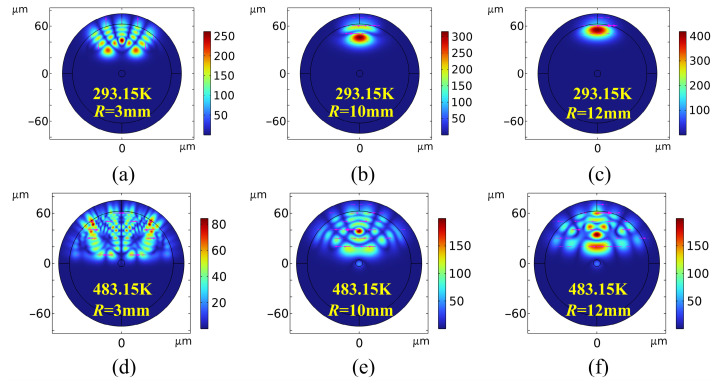
A comparison of electromagnetic field maps for bending losses in SMF at room temperature and at 483.15 K.

Figure 11 and Figure 12 present comparisons of the electromagnetic field responses at 1.55 μm. A comparison between the MMF field profiles at 293.15 K (Figure 11a–c) and at 483.15 K (Figure 11d–f) reveals that optical confinement improves significantly as the bending radius increases from *R* = 3 mm to 12 mm. This increased confinement correlates with a suppression of bending loss. In contrast, tight bending (*R* = 3 mm) induces asymmetric, multi-peak field distributions, signaling strong HOM excitation and mode coupling effects.

Comparing Figure 11a,d reveals that, at 483.15 K, the peak core field intensity decreases from 200 to 140 V/m. This attenuation expands the mode field and increases field intensity at the core–cladding interface. At a constant bending radius, the reduction in the high-temperature field validates an increase in thermal loss. This phenomenon is attributed to an intrinsic mismatch between the TOCs (d*n*/d*T*) of the core and cladding materials. Thermal excitation exacerbates this discrepancy, leading to a sharp decrease in the relative refractive index difference, Δ (Equation (13)). This thermally induced degradation of the refractive index profile directly weakens the waveguide’s lateral confinement, thereby accelerating energy dissipation into cladding radiation modes [44].

(13) $$\Delta =\frac{n_{\mathrm{core}}^{2}-n_{\mathrm{clad}}^{2}}{2n_{\mathrm{core}}^{2}}\approx \frac{n_{\mathrm{core}}-n_{\mathrm{clad}}}{n_{\mathrm{core}}}$$

Furthermore, the physical characteristics of bending loss are governed by the critical bending radius (*R*_c_). Rising temperatures reduce the fiber’s effective numerical aperture (NA_eff_), consequently increasing the critical radius *R*_c_ [45]. An increased *R*_c_ implies that, at a fixed bending radius (*R*), numerous HOMs satisfy the criteria for radiation-mode conversion, thereby accelerating the transition of guided modes into radiation modes. This mechanism drives significant evanescent field penetration into the cladding, manifesting macroscopically as the enhanced leakage intensity observed in Figure 11d–f.

Figure 12 presents a comparison of the bending radiation field evolution in SMFs under ambient (293.15 K) and extreme high-temperature (483.15 K) conditions. Unlike the complex HOM coupling in MMFs, the loss mechanism in SMFs primarily involves direct radiation leakage from the fundamental mode into the cladding. In the room-temperature group (Figure 12a–c), despite severe leakage at *R* = 3 mm, the SMF maintains strong waveguide confinement at larger radii (*R* ≥ 10 m, with peaks increasing from 250 to 400 V/m). In this regime, it exhibits only a typical centrifugal mode field shift induced by geometric bending and elastic scattering [46].

However, at 483.15 K (Figure 12d–f), the topological field distribution differs markedly from room-temperature observations. As shown in Figure 12e,f, even under large bending radii (*R* ≥ 10 mm), the fundamental mode—previously in a steady state—undergoes severe asymmetric distortion and energy dissipation (peaking at 150 V/m), while complex, large-area interference fringes are excited in the cladding. This confirms the dominant role of the Thermo-Optic Effect in SMF bending loss: extreme temperatures exacerbate the degradation of core and cladding refractive indices, significantly reducing the relative refractive index difference and the mode confinement factor. Crucially, the cladding oscillations in Figure 12 represent intense spatially coherent interference rather than the excitation of higher-order guided modes. This interference occurs between leaking radiation waves or between the radiation field and the residual fundamental mode. This severe radiation coupling and phase transition, driven by dual thermo-mechanical perturbations, elucidates the physical mechanism underlying the nonlinear signal drop and distortion observed in extreme environments such as high-temperature deep wells.

### 4.2. Analysis of the Parametric Domain of the Temperature Response of Fiber Macro-Bend Loss

**Figure 13:**
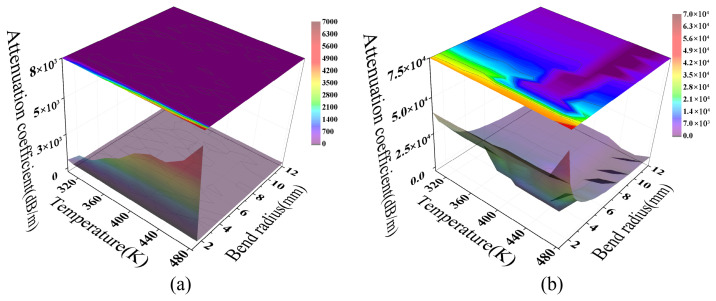
The temperature response of macro-bend loss for (**a**) MMF and (**b**) SMF.

Figure 13 illustrates the coupled effects of temperature and macro-bending strain on fiber attenuation at a constant wavelength of 1.55 μm. At small bending radii, macro-bend losses for both fiber types increase sharply with curvature, reaching magnitudes of 10^3^ for MMF and 10^4^ for SMF. This indicates that bending-induced mode leakage is the dominant mechanism in this regime. Concurrently, the TOE modulates the refractive index profile, further influencing the evolution of transmission loss.

The loss topologies of the two fiber types exhibit significant differences. For MMF (Figure 13a), the loss surface is notably smoother, a characteristic attributed to statistical averaging in multimode systems. In this model, the attenuation coefficient is derived from the Poynting vector integral over the core cross-section, representing total guided power dissipation under full-mode excitation. Because propagation constants and radiation characteristics vary across modes, interference during multimode superposition tends to cancel out on a macroscopic scale. This suppresses local oscillatory structures, yielding a relatively smooth loss surface evolution within the parameter space [47].

In contrast, the SMF loss surface (Figure 13b) exhibits distinct non-monotonic fluctuations. This is primarily due to fundamental mode coupling with radiation or leakage modes during bending. The accompanying interference effects increase the sensitivity of energy leakage to parameter variations. Temperature further modulates the effective optical path length (OPL) by altering the refractive index distribution (d(OPL) ≈ *L*d*n*/d*T*), shifting the loss features continuously within the parameter space. The loss extrema exhibit a distinct diagonal distribution relative to the temperature and bending axes. This reflects the migration of interference conditions as the optical path difference varies.

The results in Figure 13 demonstrate that the model effectively distinguishes between SMF and MMF response mechanisms under coupled macro-bending and temperature effects. Specifically, SMF exhibits structured modulation dominated by finite coupling channels, whereas MMF shows a smooth response due to multimode statistical averaging. This distinction provides the basis for frequency- and phase-domain analyses. Furthermore, it confirms the model’s physical consistency in describing bending loss across different fiber types from a parameter space perspective.

### 4.3. The PSD and Phase Analysis of the Temperature Response of Fiber Macro-Bend Loss

**Figure 14:**
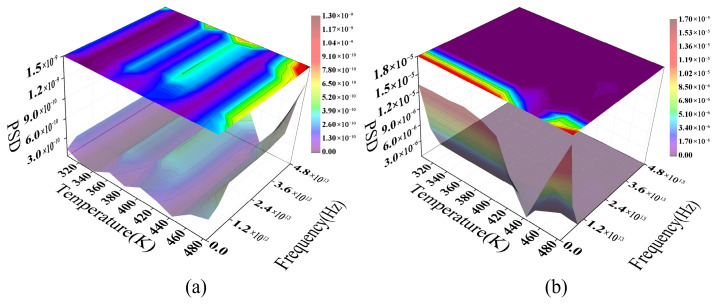
The temperature-dependent PSD of fiber bending loss: (**a**) MMF; (**b**) SMF.

To elucidate the modulation mechanisms of macro-bend loss, this study applies a Fast Fourier Transform (FFT) to MMF and SMF loss data at 1.55 μm across a coupled range of temperature (303.15~483.15 K) and bending radius (1~12 mm). The resulting three-dimensional PSD maps are then plotted. Note that the frequency axis in Figure 14 represents parameter-domain frequency rather than optical- or time-domain frequency. This axis is derived by applying a Fourier transform to the relationship between the attenuation coefficient and the external parameters (temperature and bending radius) shown in Figure 13. This frequency characterizes the loss curve’s variation scale within the external parameter space, effectively representing the “fluctuation rate” relative to temperature or geometric perturbations. Consequently, the PSD peak positions reflect the dominant characteristic modulation scales of the system response rather than actual physical oscillation frequencies.

The PSD distribution for the MMF (Figure 14a) exhibits a continuous, spreading “ridge-like” structure, suggesting that multimode interference drives the dominant modulation process. Waveguide geometric distortions induced by macro-bending facilitate energy coupling between the fundamental mode and HOMs. Due to differing effective refractive indices, these modes accumulate phase differences during propagation, resulting in a continuous interference pattern within the frequency domain. As temperature increases, the TOE subtly modifies the refractive index profile and mode field distribution. This causes spectral peaks to drift continuously as their amplitudes simultaneously adjust. This behavior aligns with the mode coupling and dispersion-induced interference typically observed in multimode systems.

In contrast, the PSD characteristics of SMF (Figure 14b) are significantly more coherent. SMF supports only fundamental-mode propagation, where macro-bending primarily induces energy leakage by introducing radiative mode coupling channels. This interference stems from the coherent superposition of the fundamental mode and the leaked radiative field over a finite path. Consequently, the spectrum exhibits a relatively narrowband, stable primary frequency component. Temperature variations drive a nearly continuous drift in this characteristic frequency by modulating the effective refractive index and leakage conditions, while the overall spectral profile remains stable.

Comparison reveals that MMF and SMF exhibit significantly different loss modulation pathways under coupled macro-bending and thermal conditions. The former is dominated by distributed coupling and inter-modal interference, resulting in a broadened, multi-peaked spectrum. In this case, the PSD main peak amplitude increases by over an order of magnitude, displaying distinct structural features. Conversely, the latter is restricted to a single dominant coupling channel, yielding a more concentrated frequency response. This distinction validates the model’s ability to differentiate between multimode and single-mode bending loss mechanisms from a frequency-domain perspective.

**Figure 15:**
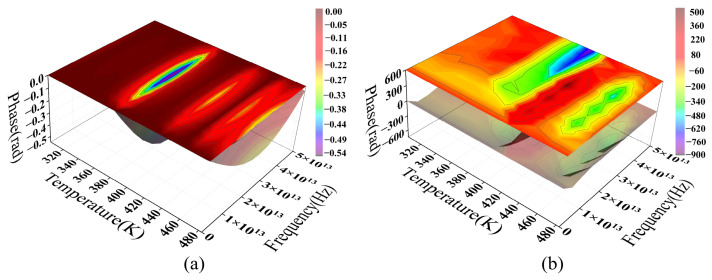
The temperature-dependent phase spectra of macro-bend loss for MMF and SMF: (**a**) MMF model data; (**b**) SMF model data.

Figure 15 further reveals the divergent phase responses of the two fiber types under coupled thermo-mechanical (macro-bending) effects. The MMF phase spectrum (Figure 15a) exhibits distinct discontinuities in the parameter-frequency space, with rapid transitions in localized regions, attributable to the coherent superposition of multimode fields. Specifically, the accumulated phase evolution of various propagation modes differs under thermal and bending perturbations. When the composite field amplitude approaches a minimum, phase sensitivity to weak perturbations rises sharply, leading to abrupt phase behavior. This phase discontinuity represents the mapping of multimode interference within the parameter domain. Although analogous to the phase singular response near a complex amplitude of zero, it is not a spatial topological singularity in the strict sense [48].

For SMF, the phase spectrum exhibits a regular periodic structure. Its sawtooth-like features arise from phase wrapping within the [−π,π] interval, reflecting continuous phase evolution relative to external parameter changes. This response is dominated by coupling between the fundamental mode and bending-induced radiation modes. Due to the limited number of interference channels, the system exhibits high stability and repeatability. Temperature variations modulate the effective refractive index via the TOE, inducing a smooth phase drift relative to the external parameter:

(14) $$\phi =\frac{2\pi }{\lambda }\cdot \Delta n_{\mathrm{eff}}(T)\cdot L$$

In comparison, the MMF phase response is driven by multimode coupling, leading to increased nonlinearity and instability. Conversely, the simpler modal structure of SMF results in a more deterministic phase evolution. This phase-domain distinction further confirms that these two fiber types exhibit divergent loss modulation mechanisms under macro-bending and TOE conditions.

### 4.4. The Wavelength-Domain Analysis of the Temperature Response of Fiber Macro-Bend Loss

**Figure 16:**
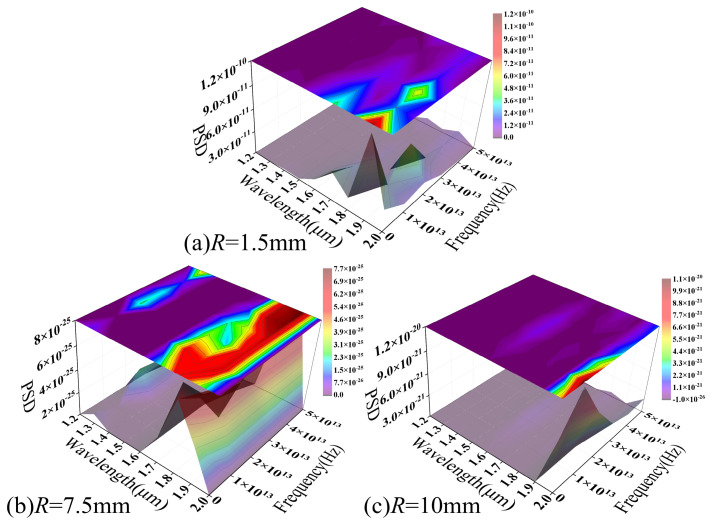
The PSD of macro-bend loss–temperature response versus wavelength in the MMF model.

Figure 16 illustrates the frequency-domain characteristics of the MMF macro-bend loss–temperature response across the 303.15–483.15 K range, plotted as a function of the operating wavelength. As the bending radius increases from 1.5 to 10 mm, the overall PSD amplitude drops from 10^−10^ to 10^−20^. This decline approaches the numerical resolution limit, indicating a progressive attenuation of both bending-induced mode coupling and temperature modulation effects.

At a small bending radius (Figure 16a), the PSD exhibits a well-defined structured distribution in the wavelength–frequency plane, characterized by continuous spectral peaks and “ridge-like” features. As the operating wavelength increases, the dominant spectral peaks shift collectively toward lower frequencies. This reflects the scaling of interference features within the parameter space, attributable to the inter-mode phase difference (Δ*β*). Wavelength variations adjust the propagation constant differences between modes, altering the spatial and frequency distributions of interference features and causing a systematic shift in spectral peak positions. Concurrently, the spectral peak contrast increases in the long-wavelength region, likely stemming from weakened mode confinement and a heightened sensitivity to curvature and refractive index perturbations. Furthermore, the local weakening or splitting of the spectral structure in specific wavelength ranges reflects fluctuating modal contributions during the coupling process. This illustrates the complex mode evolution governed by coupled macroscopic curvature and temperature effects.

As the bending radius increases (Figure 16b,c), the PSD structure blurs and the spectral peaks fade significantly. This indicates that, at larger bending radii, bending-induced mode coupling weakens significantly. The system response thus shifts from dominant local interference to a gradual, global modulation. Figure 16 reveals a distinct wavelength dependence in the MMF macro-bending loss–temperature response from a frequency-domain perspective. The modulation intensity is governed by both bending geometry and the degree of mode coupling. This conclusion is corroborated by the previously discussed loss distribution and phase spectral analyses, and further validates the model’s physical accuracy in describing multimode macro-bending response characteristics.

**Figure 17:**
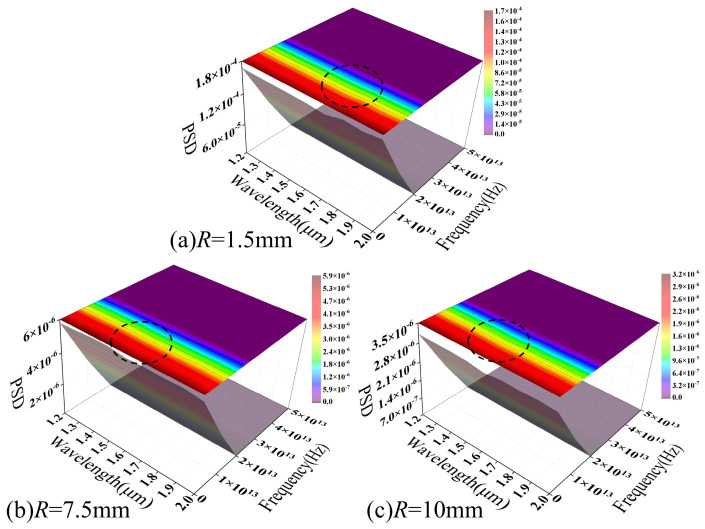
The PSD of macro-bend loss–temperature response versus wavelength in the SMF model.

Figure 17 illustrates the PSD distribution of SMF macro-bend loss as a function of wavelength across various bending radii. A continuous “main ridge” (indicated by the dashed line) persists across the wavelength dimension of the spectrum for all bending radii, with the standard deviation of its frequency axis offset remaining below 20%. This suggests that, under coupled macro-bending and thermal effects, SMF loss modulation is governed by a single dominant mechanism. Furthermore, the corresponding characteristic modulation scale remains stable across the examined parameter space.

As the bending radius increases from 1.5 mm to 10 mm, the main ridge amplitude decreases from 10^−4^ to 10^−6^, whereas its overall structure and position remain virtually unchanged. Increasing the bending radius primarily attenuates the intensity of bending-induced mode-to-radiation mode coupling without altering the dominant modulation mechanism. This behavior contrasts sharply with that of MMFs, where spectral broadening and structural dissipation occur as the radius increases. This further highlights the inherent stability of the single-mode response mechanism.

Along the wavelength axis, the main ridge exhibits a continuous and smooth evolution. The characteristic scale of loss modulation adjusts progressively with wavelength changes, stemming from mode field expansion induced by wavelength variations. This expansion alters the optical field’s sensitivity to curvature and refractive index perturbations, thereby modulating the coupling efficiency of radiation modes and their frequency-domain responses. Under coupled macro-bending and temperature effects, the SMF exhibits stable modulation characteristics centered on a single dominant coupling channel. The spectral structure manifests primarily as a narrowband response with concentrated energy rather than a superposition of multiple scales, further validating the model’s physical consistency in describing SMF bending loss from a frequency-domain perspective.

**Figure 18:**
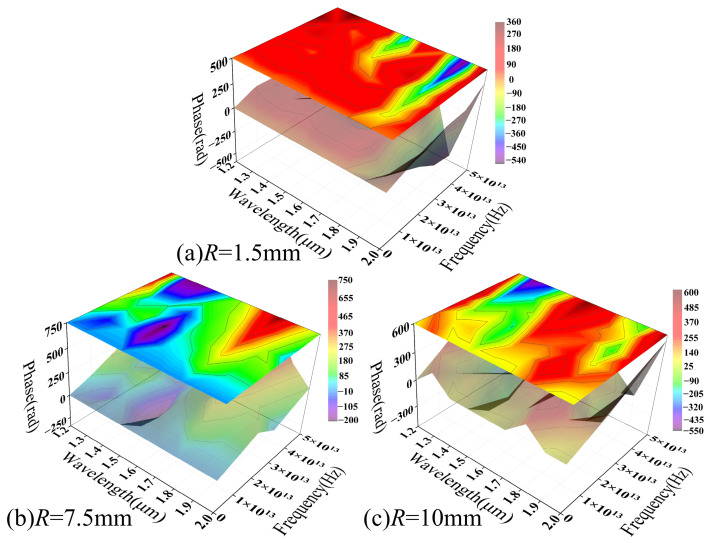
The phase spectrum of macro-bending loss–temperature response as a function of wavelength in the MMF model.

Figure 18 illustrates the phase spectrum evolution within the parameter range corresponding to Figure 16. Notably, all phase distributions have undergone phase unwrapping to demonstrate continuous phase evolution across the varying parameters. At small bending radii (Figure 18a), the phase spectrum exhibits a broad and relatively smooth gradient distribution that coincides with high-amplitude PSD regions, specifically around 1.5 μm and 1.8 μm, indicating a high SNR that allows the loss modulation to be stably characterized in the phase domain.

For larger bending radii (Figure 18b,c), the phase spectrum gradually exhibits discontinuous distributions and local “island-like” structures. This shift corresponds to a significant reduction in PSD amplitude, suggesting that, as the signal approaches the numerical resolution limit, the phase becomes hypersensitive to minute perturbations, resulting in unstable characteristics. Consequently, these phase fluctuations likely reflect numerical uncertainty driven by a declining SNR rather than a distinct physical modulation process.

Mechanistically, significant mode field distortion at small bending radii enhances the coupling between guided modes and radiation modes. This allows thermal-induced refractive index perturbations to be effectively converted into phase modulation. Conversely, increasing the bending radius strengthens mode confinement and weakens bending-induced coupling, thereby reducing the overall modulation response. These trends indicate that the MMF phase response under coupled macro-bending and thermal effects exhibits a distinct dependence on the bending radius. Its detectability is constrained by both modulation intensity and the SNR. Figure 18 corroborates the relationship between the modulation intensity and the bending radius shown in Figure 16 from a phase-domain perspective. At small bending radii, the system response is readily resolvable in both the frequency and phase domains, while at larger radii the response flattens, and its features are gradually obscured by the noise floor.

**Figure 19:**
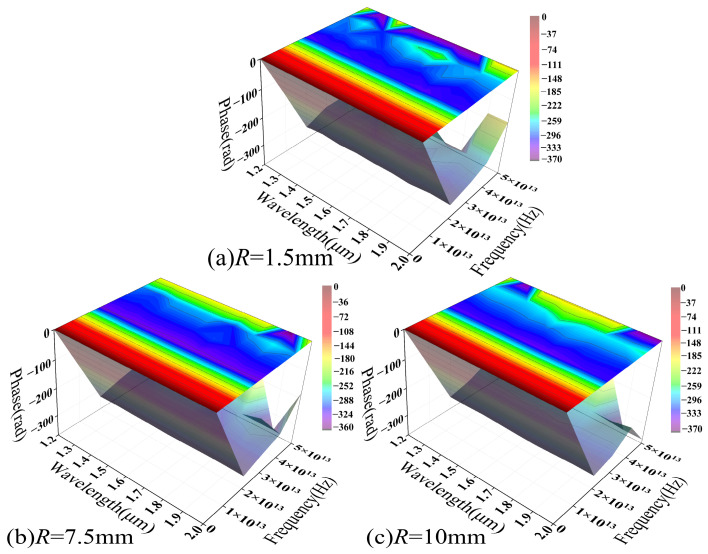
The phase spectrum of macro-bending loss–temperature response as a function of wavelength in the SMF model.

Figure 19 illustrates the wavelength-dependent evolution of the SMF phase spectrum across various bending radii. Within the range of *R* = 1.5 mm to 10 mm, the phase distribution maintains a continuous, smooth, and band-like gradient, with fluctuations of approximately 10%. The overall morphology remains highly consistent across all examined radii, showing no evidence of phase discontinuities or localized abrupt changes characteristic of MMF.

A comparison with the PSD results in Figure 17 shows that, although the macro-bending loss amplitude drops from 10^−4^ to 10^−6^ as the bending radius increases, the overall phase spectrum structure remains significantly stable. The phase distribution maintains high continuity and distinguishability across various bending conditions, indicating that the system response remains above the numerical uncertainty threshold within the current parameter range. Consequently, the phase information reliably reflects trends in external parameters.

Along the wavelength axis, the phase exhibits a smooth and regular evolution, reflecting the thermal modulation of the effective refractive index and propagation constant via the TOE. This process induces continuous phase changes. In the absence of random phase superposition from multimode coupling, the system demonstrates high stability and reproducibility within the parameter space.

Figure 18 and Figure 19 illustrate the phase spectral distributions of MMF and SMF under identical parameters. A comparison reveals that their phase responses under coupled macro-bending and thermal effects exhibit strikingly different characteristics. Overall, the phase-domain distinction between MMF and SMF primarily lies in response stability and resolvability. While the former, driven by multimode coupling, exhibits significant modulation at small bending radii, its phase structure is sensitive to parameter variations and tends to degrade under weak modulation. Conversely, the latter maintains stable, continuous phase evolution across a broad parameter range due to its single-mode transmission characteristics. These phase-domain results further confirm the fundamental differences in macro-bending and thermal loss modulation mechanisms between the two fibers. Combined with the frequency-domain analysis, these findings provide a coherent physical framework.

## 5. Conclusions

In this study, we investigate the thermal response of macro-bending loss in optical fibers under high-temperature conditions, unifying bending and TOEs as equivalent refractive index perturbations. A numerical model is constructed where bending dominates and temperature serves as the modulating factor, with macro-bending loss being subsequently calculated under full-mode excitation based on the characteristic frequency. Building on this framework, we systematically compare the response characteristics of MMF and SMF across three dimensions: the spatial, frequency (PSD), and phase domains. This work establishes a unified physical framework for interpreting these multi-domain responses.

Within the examined parameter range, the temperature-induced modulation of macro-bend loss is primarily driven by refractive index variations, with a TOC (~10^−5^ K^−1^) that is consistent with literature values. Although macro-bend losses in both fiber types scale rapidly with increased curvature, their topological characteristics differ significantly. MMF exhibits a generally smooth loss distribution, reflecting the statistical averaging of its multiple modes, while SMF shows non-monotonic fluctuations, indicating a high sensitivity to bending and thermal perturbations within finite propagation channels.

Frequency-domain analysis reveals that MMF possesses structured PSD characteristics under tight bending. As the bending radius increases, spectral intensity decays toward the numerical resolution limit (~10^−20^), suggesting that mode coupling strength dominates its modulation capability. In contrast, SMF maintains a continuous and stable spectral structure. Although its amplitude decreases from 10^−4^ to 10^−6^ as the bending radius increases from 1.5 to 10 mm, its parametric consistency suggests a modulation process governed by finite coupling paths. The phase spectra further highlight fundamental differences in stability: MMF achieves substantial phase modulation under tight bending (*R* = 1.5 mm) but exhibits discontinuities in weak modulation regions (*R* ≥ 10 mm), indicating a sensitivity to signal amplitude. Conversely, SMF maintains a smooth phase gradient across all bending radii, demonstrating superior stability and resolvability. Finally, wavelength-domain analysis reveals that MMF frequency characteristics are highly wavelength-dependent, with significant structural shifts between 1.2 and 2.0 μm, whereas SMF fluctuations remain below 10%, demonstrating greater parametric robustness.

These findings provide significant guidance for fiber selection and system design in high-temperature downhole telemetry. SMF exhibits continuous and stable response characteristics across a broad range of bending radii, making it ideal for telemetry and communication links requiring high signal integrity. Conversely, the spectral and phase responses of MMF show limited stability within the weak modulation regime, indicating its suitability for short-distance links with less complex signal architectures. Furthermore, MMF exhibits significant wavelength dependence, whereas SMF’s spectral characteristics remain stable. This suggests that the impact of environmental coupling can be mitigated by optimizing the operating wavelength.

Despite the contributions of this study, we have employed scalar approximation and do not explicitly account for polarization-dependent loss (PDL). Although bending-induced birefringence introduces minor loss deviations between orthogonal polarization states, the isotropic thermo-optic response ensures that fundamental temperature-sensitivity trends remain consistent across all polarizations. Furthermore, while this steady-state numerical model provides a robust theoretical foundation, it does not yet incorporate dynamic mechanical loads, chemical corrosion, or transient thermo-mechanical coupling inherent to complex downhole environments. In HTHP conditions, thermal expansion mismatches between the coating and the quartz substrate can induce supplemental stresses and birefringence, potentially compromising long-term stability. Consequently, future research should transition toward full-vectorial, multi-physics models that integrate mechanical, thermal, and optical fields. Calibrating these models with experimental and field data and conducting time-domain analyses to evaluate dynamic responses will further refine the model’s precision and enhance its engineering applicability for deep-well fiber-optic telemetry.

## Figures and Tables

**Table 1:** The parameters of the models.

Physical Quantity	Value	Description
*λ*	1.2–2.0 μm	Wavelength
*R*	1–12 mm	Bending radius
*T*_1_	303.15~483.15 K	Operating temperature
$n_{clad}^{s}$	1.4565	Initial refractive index of SMF cladding
$n_{\mathrm{core}}^{s}$	1.461	Initial refractive index of the SMF core
$n_{clad}^{m}$	1.4565	Initial refractive index of MMF cladding
$n_{\mathrm{core}}^{m}$	1.48	Initial refractive index of MMF core
*d*_1_	$1.2\times 10^{-5}$ K^−1^	TOC of SMF core
*d*_2_	$1.0\times 10^{-5}$ K^−1^	TOC of SMF/MMF cladding
*d*_3_	$1.5\times 10^{-5}$ K^−1^	TOC of MMF core
*R*_core1_	25 μm	MMF core radius
*R*_core2_	4.15 μm	SMF core radius
*R*_clad_	62.5 μm	Fiber-optic cladding radius

## Data Availability

The data presented in this study are openly available in OF-macrobending-T https://github.com/08blackhorse/OF-macrobending-T (accessed on 21 April 2026).
